# The Effects of Olive Leaf Extract on The Testis, Sperm Quality and Testicular Germ Cell Apoptosis in Male Rats Exposed to Busulfan

**DOI:** 10.22074/ijfs.2019.5520

**Published:** 2019-01-06

**Authors:** Sepideh Ganjalikhan Hakemi, Fariba Sharififar, Tahereh Haghpanah, Abdolreza Babaee, Seyed Hassan Eftekhar-Vaghefi

**Affiliations:** 1 Department of Anatomy, Afzalipour Faculty of Medicine, Kerman University of Medical Sciences, Kerman, Iran; 2 Herbal and Traditional Medicines Research Center, Department of Pharmacognosy, Faculty of Pharmacy, Kerman University of Medical Sciences, Kerman, Iran; 3 Department of Anatomy, Kerman Branch, Islamic Azad University, Kerman, Iran

**Keywords:** Apoptosis, Busulfan, Olive Extract, Spermatogenesis, Testicular Germ Cell

## Abstract

**Background:**

Busulfan (BU) has a destructive effect on the male reproductive system. The goal of this study was to assess the effects of olive leaf extract (OLE) as a source of antioxidants and phenolic compounds, on BU-induced damages in rat testes.

**Materials and Methods:**

In this experimental study, 40 male Wistar rats were randomly divided into 5 groups. The control group (CTL) received a single intraperitoneal (i.p.) injection of dimethyl sulfoxide (DMSO), followed by oral administration of distilled water for 5 weeks. In BU group, BU (10 mg/kg) was administrated i.p. once. In co- treatment groups, first, received BU (10 mg/kg, a single i.p. injection) then, OLE was administrated orally at different doses of 250 mg/kg (BU+OLE 250), 500 mg/kg (BU+OLE 500) and 750 mg/kg (BU+OLE 750), for 5 weeks. Next, blood and sperm samples were collected. The left testis was removed to investigate testicular parameters and apop- tosis by using H&E and TUNEL staining, respectively. All data were analyzed by SPSS software and a P<0.05 was considered significant.

**Results:**

There was a significant decline in sperm viability (P=0.017), number of primary spermatocyte (PS) (P=0.001) and Leydig cells (P=0.023) in the BU group versus the CTL group. OLE at three doses could repair these defects ver- sus BU group. Increases in apoptotic spermatogonia cells (SG) due to BU were significantly reduced by OLE 250 and 500 mg/kg (P<0.01). A reduction in germinal epithelium height and an increase in apoptotic SG were observed in BU+OLE 750 group vs. other groups (P<0.01) and alkaline phosphatase (ALP) was at the highest level, also Aspartate aminotransferase (AST) increased markedly vs. CTL (P=0.024).

**Conclusion:**

Oral administration of OLE at the doses of 250 and 500 mg/kg could be helpful in ameliorating BU- induced toxicity in rat testes, while OLE 750 mg/kg not only did not cause positive effects, but also could exacerbate the harmful effects.

## Introduction

The use of anti-cancer drugs has been increased. Busulfan (BU) is one such anti-cancer drug that is used to treat lymphoma, chronic leukemia, and ovarian cancer. It is also used as a part of a regimen administered before bone marrow transplantation. However, studies showed that this drug has side effects on many organs such as the male reproductive system (1). The negative effects of BU on the male reproductive system include decreasing testis weight (2), increasing abnormal sperm parameters (motility and morphology) (3), oligo-azoospermia, destroying almost all testicular germ cells (4), and causing temporary or permanent sterility.

Since BU is an alkylating agent with oxidative properties (5), therefore, it is believed that antioxidant therapy may be helpful in reducing its harmful effects. Several animal studies have reported an ameliorating effect of plant extracts possessing antioxidant properties on the male reproductive system following exposure to BU (1, 6).

Olive (*Olea europaea L.*) is commonly used as a part of traditional herbal medicine to treat disease in the Mediterranean area (7). Olive leaf is rich in antioxidant phenolic compounds such as oleuropein, verbascoside, ligstroside, as well as flavonoid compounds like tyrosol and hydroxytyrosol (7, 8). Oleuropein scavenges harmful free radicals and prevents oxidative damage (9). It was reported that treatment with olive leaf extract (OLE) improved total antioxidant capacity (TAC) level in rat testicular tissue. Also, it was shown that OLE can improve sperm parameters and testis antioxidant conditions in rats exposed to rotenone (10).

With increasing prevalence of cancer, the number of individuals being treated with BU has significantly increased. Noteworthy, most of these BU-treated subjects are in childbearing ages and it is not possible to restore fertility following BU exposure. Therefore, research on new agents and/or herbal extracts which can reduce these adverse effects on the male reproductive system is essential. For the first time, in this study, OLE which contains phenolic compounds and exerts antioxidant properties, was given to different groups of BU-treated animals to investigate the effect of oral administration of OLE on testis structure, sperm parameters and apoptosis in rat testes. To evaluate the safety profile of the extract, we measured levels of liver enzymes to assess possible toxic effects of different doses of OLE on the liver as an important organ that in involved in drug absorption and elimination.

## Materials and Methods

The present experimental study was approved by the Ethics Committee of Kerman University of Medical Sciences, Kerman, Iran (IR.KMU.REC.1394.641).

### Olive leaves extract preparation

Olive leaves were collected from the olive tree farms from Kazeroon, Iran, authenticated by an expert and kept at the herbarium of pharmacognosy department, faculty of pharmacy, Kerman University of medical sciences, Kerman, Iran. The leaves were washed and dried at room temperature. Dried leaves (500 g) were milled and passed through a sieve (mesh 300). Plant extraction was performed using warm maceration with ethanol 80% for 72 hours. Obtained extract was concentrated under vacuum and finally dried in an oven at 40°C for 24 hours. The extract was stored at -20°C for subsequent experiments. The extract was dissolved in distilled water before use (11).

### Determination of total phenol content of olive leaf extract

Total phenolic content of OLE was determined by Folin- Ciocalteu assay. Gallic acid was used for calibration. A stock solution of gallic acid (1000 ppm) was prepared; next, 0.1 ml stock solution was added to 0.4 ml sodium carbonate, 0.5 ml Folin reagent and 3 ml distilled water after 40 minutes incubation at room temperature. Absorbance was measured at 765 nm. The calibration curve was plotted for gallic acid based on 7 serial dilutions. After measurement of absorbance, calibration curve was plotted. By determination of extract absorbance as mentioned above and using the curve equation, total phenolic content was expressed as mg gallic acid equivalents per g of the extract (12). Each experiment was done in triplicate.

### Estimation of total flavonoid content of olive leaf extract

Total flavonoid content was measured by the aluminum chloride colorimetric assay. Rutin as the major flavonoid compound of the plant, was assessed for standardization using thin layer chromatography. First, 1 ml rutin (50 ppm) was added to 1 ml aluminum chloride 2%. After 30 minutes of incubation at room temperature, absorbance was recorded at 200-400 nm and maximum wavelength was 275 nm. Calibration curve was prepared using different dilutions of rutin. As mentioned above , total flavonoid content of the plant was expressed as mg rutin equivalents per g of the extract (12). Each experiment was done in triplicate.

### Animals and chemicals

Adult male Wistar rats (8-10 weeks old) were obtained from animal house of the university. Animals were kept in a temperature-controlled room (at 22°C) with 12 hours/12 hours light/dark cycles. Food and water were readily available. All chemicals were purchased from Sigma-Aldrich, unless otherwise noted. 

### Experimental design

Forty adult rats were randomly divided into 5 groups of control (n=8), BU (n=9) and BU co-administrated with three doses of OLE 250 mg/kg (n=8), 500 mg/kg (n=6) and 750 mg/kg (n=8) (BU+OLE 250, BU+OLE 500 and BU+OLE 750, respectively). In this study, BU was diluted in dimethyl sulfoxide (DMSO) and distilled water (D.W.) as solvent. The OLE was dissolved in the D.W. The animals in the control group (CTL) received a single intraperitoneal (i.p.) injection of BU solvent (i.e. DMSO+D.W.) and then D.W. was administrated orally by gavage for 5 weeks. The BU group received a single i.p. injection of BU (10 mg/kg) (13), the BU+OLE 250 group received OLE (250 mg/kg) orally for 5 weeks after receiving a single BU injection (10 mg/kg, i.p.), the BU+OLE 500 group received OLE (500 mg/kg) orally for 5 weeks after receiving a single BU injection (10 mg/kg, i.p.), the BU+OLE 750 group received OLE (750 mg/kg) (14) orally for 5 weeks after receiving a single BU injection (10 mg/kg, i.p.). Some rats died during the study, especially in the BU+OLE 750 group. At the beginning and end of the experiment, all rats were weighted.

### Sample collection

After the end of the treatment period, the rats were deeply anesthetized by chloral hydrate (400 mg/kg) (15). After making an incision in their chests, the heart blood samples were collected from the left ventricle for biochemical and hormone analysis. The left testis and vas deferens were removed and separated from surrounding tissue. The testes’ weight and diameter were recorded and then tissues were fixed in formalin 10% (16) for histologic analysis. Left vas deferens was dissected and sperms were collected.

### Serum testosterone and liver enzyme levels measurement

The blood (1.5 ml) collected from the heart was centrifuged at 3000 rpm for 30 minutes. Serum was carefully separated from plasma and immediately stored in a freezer at -20°C until analyzed. The level of serum testosterone and liver enzymes including alkaline phosphatase (ALP), alanine aminotransferase (ALT) and aspartate aminotransferase (AST) were measured in duplicate samples by an enzyme-linked immunosorbent assay (ELISA) using IBL kit (IBL company, Germany) and Biorex kit (Biorex company, UK) , respectively, according to the manufacturer’s instructions. 

### Assessment of sperm parameters

Under sterile conditions, the inferior part of rat abdomen was incised, the left vas deferens was removed and placed in a petri dish containing pre-warmed alpha MEM medium (2 ml), supplemented with 10% bovine serum albumin. It was dissected into several fragments and then, incubated at 37°C with CO_2_ 5% in humidified air for 30 minutes to permit the migration of all spermatozoa from the reproductive duct to supplemented medium (15). Thereafter, the medium containing spermatozoa was collected and sperm quality was evaluated in terms of sperm motility, count, morphology and viability according to WHO guideline (17).

### Sperm motility

Immediately, 10 µl of sperm suspension (including the supplemented medium and spermatozoa) was placed on a slide and covered by a coverslip. Sperm motility was classified as fast progressive motility, slow progressive motility and immotile according to WHO guideline (17) and expressed as the percent of each one per total sperm number in at least 10 fields (300 sperms).

### Sperm count

First, 10 µl of sperm suspension was added into 10 µl of fixative solution (formalin/sodium bicarbonate). Next, 10 µl of this mixture was placed on Neubauer haemocytometer and covered by a coverslip. The counting chamber was then placed on the light microscope stage (Nikon TS-100, Japan) at ×200 magnification, and sperms were counted in four large squares. The average of counted sperms was multiplied and was expressed as million/ml of suspension (18).

### Sperm viability 

Sperm viability was assessed using eosin-nigrosin staining. Sperm suspension (5 µl) was added to eosin-nigrosin stain (5 µl). Smear was then prepared and at least 200 spermatozoa were randomly counted under a light microscope (at ×400 magnification). Sperm with red or pink head considered dead sperm and non-stained sperm, with white head, considered alive (17). The percentages of live spermatozoa were noted.

### Measurement of body and testis weight

Body weights of rats were noted prior to start of experiment and 24 hours after the final day of treatment. The left testis was weighted by using a digital balance.

### Evaluation of testis histology

Left testis diameter, length and width were recorded by using standard digital calipers. In order to assess the alterations in spermatogonia cell (SG) population, histological evaluation of the rat testis was carried out (eight testis samples in each group). After testis fixation in formalin 10%, testis was dehydrated in increasing concentrations of ethanol (70, 90 and 100%) and embedded in paraffin. Five-micron thick sections of testis (at 50 µm interval) were prepared using microtome, mounted carefully and stained with hematoxylin and eosin (H<E). The slides were examined under a light microscope (Olympus/ BX51, Japan). In each section, 15 seminiferous tubules were randomly examined. Two perpendicular diameters of each seminiferous tubule (from the basement membrane to lumen) were calculated via calibrated linear scale of the Analysis software in the 10X eyepiece of Olympus microscope (18). The average of these diameters was reported. Also, thickness of the germinal epithelium layer was measured. The number of SG, primary spermatocyte (PS) cells and also Leydig cell were recorded from 10 microscopic fields at ×400.

### Evaluation of testicular apoptotic cells by using TUNEL assay 

Terminal deoxynucleotidyl-transferase-mediated DNA nick end-labelling (TUNEL) assay is a valuable method to detect apoptotic cells by labeling the terminal end of nucleic acids. TUNEL staining was done by an in situ cell death detection kit, POD (Roche-11684817910 version 14, Germany), according to the manufacturer’s instructions.

First, the testicular slides were deparaffinized by incubation at 60°C for 30 minutes, and then rehydrated in xylene (for 30 minutes) and increasing concentrations of ethanol 70, 90, and 100%, respectively (each one for 6 minutes). Slides were washed twice with distilled water. 

Next, the slides were incubated in proteinase K (20 µg/ml in 10 mM Tris buffer) at 37°C for 30 minutes and washed three times with phosphate buffered saline (PBS). Afterwards, the slides were incubated in hydrogen peroxide solution (H_2_O_2_) 3% at room temperature for 10 minutes in the dark and re-washed three times with PBS. Immediately, TUNEL reaction mixture (enzyme solution 50 IU and label solution 450 IU) was prepared. The sections were incubated in a moist chamber containing a TUNEL reaction mixture at 37°C temperature for 60 minutes. After three-time washing by PBS, the slides were incubated in POD (anti-fluorescein antibody, FAB fragment from sheep, conjugated with peroxidase) intra a moist chamber for 30 minutes at 37ºC. The sections re-washed three times with PBS. 3,3'-Diaminobenzidine (DAB) substrate was added to the slides and incubated at room temperature for 10 minutes in the dark. After washing with PBS (once), the slides re-washed carefully with distilled water. Next, sections were stained with hematoxylin at room temperature for 30 seconds. After washing the slide with distilled water and dehydration by ascending degrees of ethanol (70, 90 and 100% respectively), the slides were mounted using Entellan. TUNEL-positive cells per tubule in at least 20 tubules from the testes were counted under a light microscope (Olympus/BX51, Japan) (18).

### Statistical analysis

Statistical analysis was carried out by using Statistical Package for the Social Sciences software, version 21 (SPSS, Chicago, IL, USA). All data were expressed as means ± standard errors of the mean (SEM). At first, one-sample Kolmogorov-Smirnov test was used to check the normality of variables. Next, the differences in normal-distributed variables among five experimental groups were analyzed by using one-way ANOVA test followed by Tukey post hoc test. For nonparametric variables, non-parametric Kruskal- Wallis test (TUNEL SG, germinal epithelium height, alive and fast progressive sperm, and length of testis) was used. The level of significance was set at P<0.05.

## Results

### Assessment of flavonoids and phenolic content of olive leaf extract

**Fig.1:**
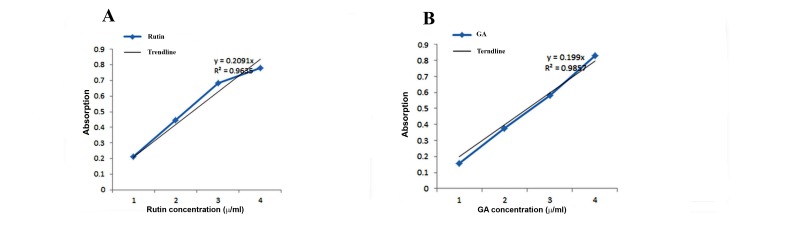
The standard curves of phenols (gallic acid equivalents) and flavonoids (rutin equivalents) by drawing adsorption against concentration. **A.** Standard curves of rutin and **B.** Standard curve of gallic acid. The results have been resulted from triplicate experiments.

The total flavonoid content of the OLE, calculated using calibration curve of rutin (R2=0.9635) was 1.43 g ruin equivalent/g plant extract. Total phenolic content of the plant, calculated from gallic acid standard curve (R2=0.9857) was 1.44 g gallic acid equivalents in 1000g OLE (Fig .1).

### Testosterone and liver enzymes assay 

There was no significant difference in blood levels of testosterone among different groups. Analysis of the liver enzyme level showed that ALP level increased significantly in BU+OLE 750 compared to control (P<0.001), BU (P<0.001), BU+OLE 500 (P=0.003) and BU+OLE (P=0.030). Also, ALP level in BU+OLE 250 was significantly elevated versus the control (P=0.011). Compared to the BU group, AST and ALT enzyme levels did not vary significantly between BU+OLE 250 and BU+OLE 500 groups. Furthermore, AST level in rats treated with 750 mg/kg OLE showed a significant increase as compared to BU (P=0.005) and CTL (P=0.024, Table 1).

### Rat body and testis weights measurement and morphological assessments

There were no significant differences in testis weight and changes in rat body weight in any group (P>0.05). The testis width, length and diameter in OLE-treated animals (all doses) remained unchanged and were similar to those of the control and BU-exposed animals (P>0.05) (data not shown).

### Sperm characteristics 

The effects of BU and different doses of OLE on sperm count, motility, morphology, and viability are summarized in Table 2. Comparing sperm count among 5 groups by using one way-ANOVA test, showed that although exposure to BU could non-significantly decrease the sperm count (3.23 ± 0.62, P=0.086) as compared to that of CTL (5.75 ± 0.82), all doses of OLE caused an increase in sperm count as compared to BU group. Fast progressive motility decreased non-significantly in BU group (4.74 ± 1.33) vs. the CTL (11.67 ± 4.97) (P=0.19).

Oral administration of OLE at different doses of 250 (17.91 ± 2.85), 500 (28.75 ± 5.86) and 750 (20 ± 4.10) mg/kg could significantly improve the proportion of sperms to the fast progressive motility versus BU group (P=0.002, P=0.009, P=0.003, respectively).

The percentage of viable sperms in the BU group was significantly lower than that of the CTL (P=0.017). Compared with BU group, a significant change was observed viable sperm percentage in all OLE-treated groups (250 mg/kg, P<0.001, 500 mg/kg, P=0.003, and 750 mg/kg, P<0.001) (Table 2).

**Table 1:** Effect of busulfan (BU) and different doses of OLE (250,500 and 750 mg/kg) on liver enzymes and testosterone hormone levels after 5 weeks of treatment


Group	ALP (U/l)	AST (U/l)	ALT (U/l)	Testosterone (ng/ml)

CTL	340 ± 41.33	155.50 ± 24.85	58.50 ±7.62	3.714 ± 1.08
BU	522.83 ± 70.19	133.25 ± 5.59	72.17 ± 5.59	3.129 ± 0.77
BU+OLE 250	648.67 ± 68.16^a^	162.80 ± 9.98	76 ± 9.98	1.63 ± 0.67
BU+OLE 500	549.20 ± 61.00	165.40 ± 10.23	73.20 ± 10.23	2.54 ± 0.89
BU+OLE 750	932.60 ±‎ 64.46‎^c^	220.83 ±‎ 6.67^a^^b^	86.67 ± 6.67	2.75 ± 0.63


Results are expressed as mean ± SEM. Significant differences (P<0.05) are indicated by ^a^; vs. control group in the same column, ^b^; vs. BU group, ^c^; BU+OLE 750 group vs. another groups in the same column, ALP; Alkaline phosphatase, ALT; Alanine aminotransferase, AST; Aspartate aminotransferase, OLE; olive leaf extract, and CTL; Control group.

**Table 2:** Effect of busulfan (BU) and different doses of OLE (250,500 and 750 mg/kg) on testis histology and sperm parameters


Group	Control	BU	BU+OLE 250	BU+OLE 500	BU+OLE 750

Spermatogonia number	37.74 ± 2.93	33.67 ± 1.82	44.35 ± 2.17^b^	42.11 ± 3.47	32.91 ± 1.93^c^
Primary spermatocyte number	174.19 ± 16.66^b^	75.52 ± 8.62^a^	161.46 ± 7.80^b^	168.42 ± 18.63^b^	128.75 ± 21.56
Leydig cell number	5.47 ± 0.43^b^	2.32 ± 0.32^a^	5.8 ± 0.92^b^	5.97 ± 1.148^b^	4.23 ± 0.71^b^
Seminiferous tubules diameter (mean D and d) (μm)	299.21‎ ± 7.68	300.48‎ ± 6.11	299.68 ± 2.88	305.66‎ ± 10.96	275.28‎ ± ‎‎7.2
Germinal epithelium height (μm)	86.29 ± 3.36	86.88 ± 2.72	131.77 ± 43.91	90.89 ± 3.63	76.66 ± 2.98^b^^c^^e^
Alive sperm (%)	48.67 ±7.51	23.61 ± ‎3.54‎^a^	57.72 ±‎ 5.60‎^b^	56.25 ±‎ 1.44‎^b^	61.72 ±‎ 6.16‎^b^
Sperm count (×10^6^/ml)	5.76 ± 0.82	3.23‎ ± 0.62^c^^f^	7.97‎ ± 0.76	6.12 ± 0.55	7.83 ± 0.74
Fast progressive sperm (%)	11.66 ± 4.96	4.74 ± 1.33	17.91 ± 2.85	28.75 ± 5.86^a^^b^	20.00 ± 4.10^b^
Slow progressive sperm (%)	43.53 ± 6.30	39.32 ± 4.94	35.25 ± 4.75	25.52 ± 5.82	38.00 ± 4.19
Immotile (%)	45.30 ± 3.65	53.94 ± 4.68	46.67 ± 3.69	45.60 ± 2.16	41.84 ± 3.04


Results are expressed as mean ± SEM. Significant differences (P<0.05) are indicated by ^a^; vs. control group in the same row, ^b^; vs. BU group in the same row, ^c^; vs. BU+OLE 250 group in the same row, ^e^; vs. BU+OLE 500 group in the same row, ^f^; vs. BU+OLE 750 group in the same row, D; Long diameter, d; Short diameter, and OLE; Olive leaf extract.

### Testis histological study

#### Spermatogenesis assessment 

Spermatogenesis in CTL testis was normal (Table 2). Although the mean number of SG in the BU-treated testis was numerically lower than the CTL, it did not reach a significant level (P=0.747). The BU+OLE 250 group showed a significant increase in the mean number of SG when compared to the BU group (P=0.026). There was a significant difference between BU+OLE 750 and BU+OLE 250 group with respect to SG number (P=0.013).

A statistically significant difference was observed in the number of the PS (P=0.001) and Leydig cells (P=0.023) between the BU and CTL. Compared to the BU group, OLE 250 (P=0.004) and 500 (P=0.003) provided a significant increase in the number of PS following BU-exposure. No statistically significant difference was observed in the average number of PS and Leydig cells between groups treated with different doses of OLE and CTL. There was a significant difference between control and BU groups with regard to the number of Leydig cells (P=0.023). The number of testicular Leydig cells in OLE 500 group was higher than that of the BU group (P=0.013).

**Fig.2:**
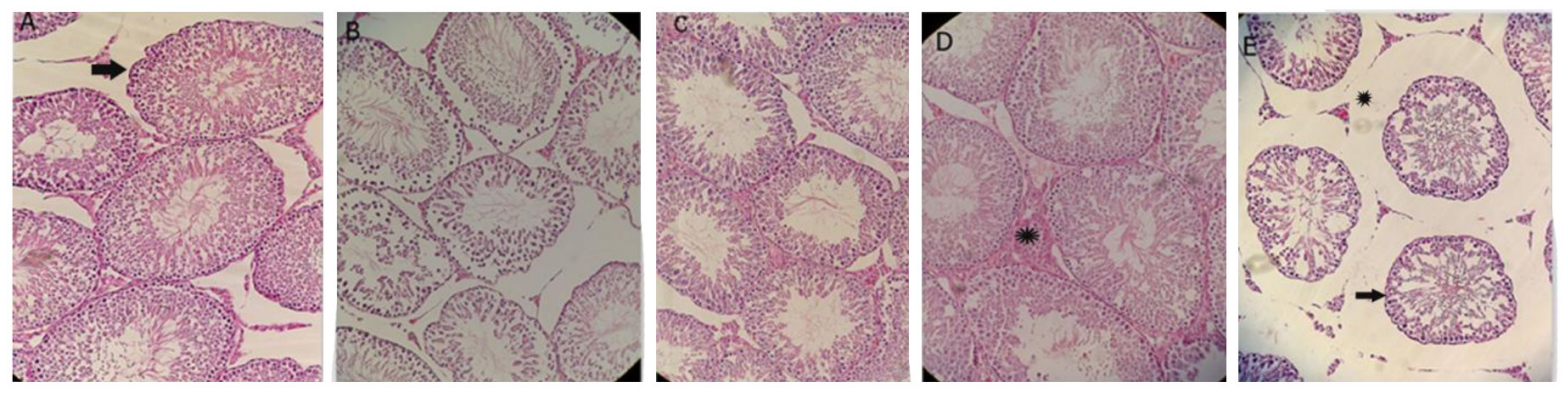
Light micrographs of rat testis (H<E staining, ×200 magnifications). **A.** Seminiferous tubules showing normal structure and active spermatogenesis in the control group, **B.** The most of spermatogonia and primary spermatocytes cells are destroyed in busulfan group, **C.** Photomicrographs of testis from rats that treated with 250 mg/kg, **D.** 500 mg/kg, and **E.** 750 mg/kg olive leaf extract (OLE) demonstrated that both 250 and 500 mg/kg OLE caused a normal and regular structure of seminiferous tubules and obvious improvement in spermatogenesis, while 750 mg/kg OLE reduced cell lines and height of seminiferous epithelium (.) and destroy interstitial space (*).

The results showed that all doses of OLE, following BU exposure, can repair spermatogenesis to varying extents (Fig .2).

**Fig.3:**
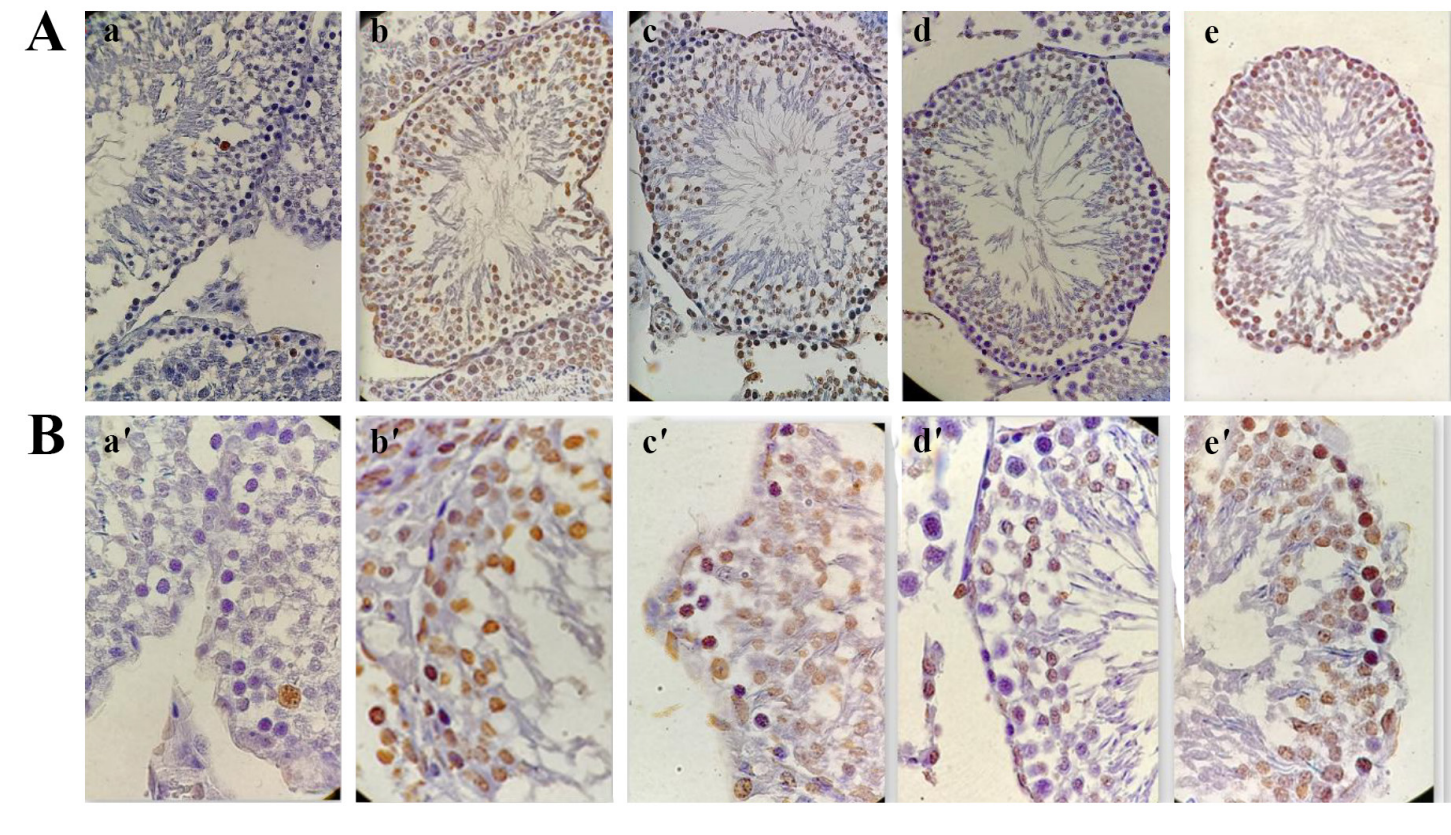
Immunohistochemical staining of the rat testis tissue in experimental groups. A. Light microscopy of TUNEL-stained rat testicular sections (×400 magnifications), a. Apoptotic cells are seen brown color. Apoptosis is extremely low in control testes, b. The most of testicular germ cells is undergoing apoptosis in busulfan testes, c. Although TUNEL-positive germ cells are still visible in testicular sections from rats that treated with olive leaf extract (OLE) at dose of 250 mg/kg, d. 500 mg/kg OLE caused a marked decrease in apoptotic testicular germ cells, e. High level of apoptotic cells was observed in testes of rats that treated with OLE at dose of 750 mg/kg and B. ×1000 magnification.

### Seminiferous tubule morphometry

No statistically significant difference was observed in the mean diameter of seminiferous tubules among different groups (P>0.05). Comparison of the mean of germinal epithelium thickness between BU-treated testis and control, BU+OLE 250 and BU+OLE 500 testes showed no significant differences (P>0.05). However, significant decreases in thickness of the germinal epithelium of BU+OLE 750 group were observed as compared to the BU (P =0.033) and OLE 250 and 500 (P=0.019) treatment groups (Table 2).

### Germ cell apoptosis assessment

**Fig.4:**
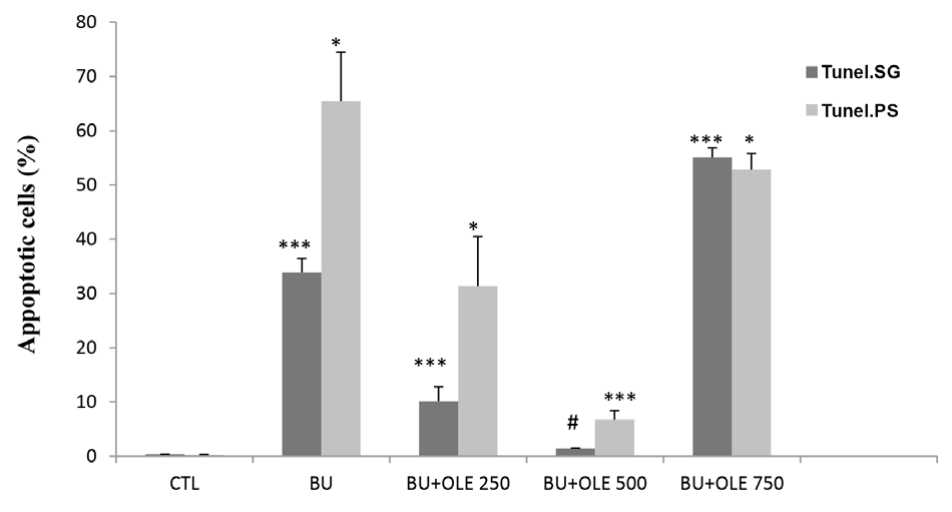
Effect of OLE treatment after busulfan exposureon percentage of apoptotic testicular germ cells. Significant differences (P<0.05) are indicated by ***; vs. all groups, *; vs. control and BU+OLE 500 groups, and #; vs. all groups except control. Values are expressed as % mean ± SEM. CTL; Control group, BU; Busulfan, PLE; Olive leaf extract, SG; Spermatogonia, and PS; Primary spermatocye cells.

In the present study, apoptotic germ cells were distinguished by TUNEL staining (Fig .3). The apoptotic index was calculated as follows: TUNEL positive nuclei (dark brown cells) /number of total germ cells. As summarized in Figure 4, a significant increase in the mean number of apoptotic SG (P<0.001) and PS (P=0.029) cells was observed in rats exposed to BU as compared the CTL. However, BU-mediated increase in apoptotic SG was significantly reduced by administration of OLE 250 and 500 mg/kg (P<0.001). This decline was markedly higher in the group administrated with OLE 500 mg/kg compared to the other doses of OLE (P<0.001), and nearer to that of the CTL. In contrast, the percentage of apoptotic SG was enhanced in rats which received OLE 750 mg/ kg, compared to the other groups (P<0.001). Also, the TUNEL-positive PS counts in the seminiferous tubules showed that OLE 250 mg/kg could not decrease the level of apoptotic PS cells significantly compared to BU group. Interestingly, only a small number of apoptotic cells were observed in the testicular sections of OLE 500 group, which was significantly different from that of the BU (P=0.029) and the other OLE groups (P=0.029). However, this reduction was more marked compared to the CTL. In contrast, the group which received OLE 750 mg/kg was shown to have significantly higher levels of apoptotic PS cells, in comparison to OLE 500 and CTL (P=0.029).

## Discussion

The present study showed that administration of a single dose of BU to Wistar rats, leads to a significant reduction in sperm and testicular parameters (i.e. sperm viability and the number of PS and Leydig cells). Furthermore, our results demonstrated that BU could increase the rate of apoptotic SG and PS in the rat testis. However, it was shown that OLE administration at two doses of 250 and 500 mg/kg to rats that received BU, could significantly improve the afore-mentioned parameters in testis following BU -induced toxicity. Oral administration of OLE at 750 mg/kg has a negative effect in many cases (i.e. the thickness of germinal epithelium, spermatogenesis lineage cells, and apoptosis), and leads to increased levels of liver enzymes. 

These findings are in line with previous reports showing toxic effects of BU in rat testis, including changes in sperm parameters and spermatogenesis along with pro-apoptotic BU potential in murine male germ cells (3, 13, 19). BU could induce oxidative damage to the testis (20). In addition to, BU is an alkylating agent that by attaching to double strand DNA could prevent DNA replication and RNA transcription leading to stem cell death. These could explain inhibition of spermatogenesis process in the present study. In the present study, sperm motility decreased non- significantly in BU-exposed rats, and also sperm tail abnormality was higher than those of the other groups. ROS can attack and damage bio-molecules such as DNA and lipids. As the sperm plasma membrane has a high content of polyunsaturated fatty acids, sperms are highly susceptible to oxidative stress. Oxidative stress induced by BU could affect the polyunsaturated fatty acids in the tail membrane of the sperm cell, disturb its fluidity and lead to a reduction in sperm motility (21). Also, previous studies showed that lenght of the sperm flagella reduces in rats that received BU, leading to decreased sperm motility (13).

In agreement with our data, Anjamrooz et al. (3) reported that sperm count, viability, and motility markedly decline after exposure to BU even at its lowest dose (10 mg/kg) and exposure period (four weeks) when compared to CTL. The destructive effect of BU on germ cell vitality was also observed. Apoptosis is programmed cell death characterized by some distinct changes in cell morphology and genetic material.

This process could occur under normal conditions of organs or abnormal situations such as chemical-induced cell death. Under normal conditions, apoptosis could happen during normal spermatogenesis to balance the ratio of germ cells and sertoli cells number in testicular tissue (22). This appropriate rate of apoptosis is most commonly seen in SG (A2, A3 and A4 stages). Abnormal conditions such as administration of cytotoxic agents, for example BU, could cause abnormal rate of apoptosis in germ cells, spermatocyte, spermatid cells and SG (23) and lead to pathological condition. 

This study, in accordance with another study (22), showed that after BU administration, the number of apoptotic SG and spermatocyte cells in rat testis tissue significantly increase. This cytotoxic agent could induce germ cell apoptosis afterward direct BU-induced damage to the germ cells by reduction of the expression level of c-kit as a survival factor, in SG (24) or indirectly via inducing apoptosis in sertoli cells (25) by increasing ck18 level (a death factor) in these testicular supporting cells. However, due to marked dependence of the germ cells on the function of the sertoli cells, the apoptosis of these supporting cells can also endanger the germ cells vitality. Also, it was suggested that BU increases malondialdehyde (MDA, a marker oxidative stress and lipid peroxidation) level. Therefore, BU-induced oxidative stress might be a reason of germ cell death, spermatogenesis disturbance and infertility (2). With increasing incidence of cancer throughout the world, the use of cytotoxic and anti-cancer drugs are increased. Therefore, many studies today focus on increasing fertility potential after exposure to cytotoxic agents. 

Nowadays, plant extracts as sources of antioxidants and phenolic compounds have attracted considerable attention. Several studies reported the improving effect of various plant extracts on BU-induced testis toxicity (1). In this study, for the first time, we examined at the effects of different doses of OLE in BU-treated animals.

OLE contains different types of polyphenolic compounds including simple phenols such as gallic acid, flavonoids such as rutin and secoiridoids such as oleuropein at different concentrations (26). Among phenolic compounds present in OLE, oleuropein, luteolin and hydroxytyrosol have powerful antioxidant activities (27). It was reported that administration of 300 mg/kg OLE markedly decreases testis MDA level and improves sperm parameters (10). In the present study, administration of OLE 250 and 500 mg/kg might cause a reduction in BU-induced ROS production, lipid peroxidation and stress oxidative in testis and therefore markedly modulated or repaired the sperm and testicular defects. Similarly, Sarbishegi et al. (10) reported that OLE 150 and 300 mg/kg (not 75 mg/ kg), improve the sperm quality and testis oxidative stress after rotenone exposure. Oleuropein, one of the phenolic constituents of olive leaf, was shown to exert ameliorating effects on alcohol-induced oxidative stress in male rat testis and improve sperm parameters (5). Inconsistent with our study, another study showed that administration of olive fruit extract to rats have a negative effect on sperm parameters (28). This might be explained by different dosage and duration of treatment.

In the current study, a decrease in the number of apoptotic germ cells also observed when OLE 250 and 500 mg/ kg were administrated to Wistar rats treated with a single dose of BU. OLE acts as an anti-apoptotic agent via decrement of the expression level of caspase 3, a death factor that could initiate apoptotic DNA fragmentation and promote apoptosis. It could also reduce the BAX/BCL2 ratio. Therefore, it seems that OLE inhibits the apoptotic pathway via reduction of pro-apoptotic proteins and improves cell vitality (29). As another mechanism, it was suggested that OLE increases antioxidant capacity due to high content of flavonoids and phenols therefore could directly scavenge free radicals (30) and/or diminish oxidative stress via increasing superoxide dismutase (SOD) and decreasing MDA levels (31). The demotion of these oxidative markers improved spermatogenesis and fertility potential.

However, OLE 750 mg/kg (the highest dose used in the present study) did not show a markedly higher efficacy compared to the other doses. Although all doses of OLE could significantly improve the number of PS and Leydig cells when compared to BU, but the numbers of spermatogenic cells and Leydig cells in the testes of the BU+OLE 750 group were not higher than those of the other doses of OLE. Also, the epithelium height in BU+OLE 750 group was the lowest. Furthermore, administration of OLE 750 mg/kg produced high apoptotic germ cell counts, even higher than BU. This data demonstrated that OLE 750 mg/kg not only failed to attenuate BU-induced testicular apoptosis but also worsened the BU impact. Similarly, several studies have shown that higher doses of herbal extracts may have adverse effects on organs. It was reported that OLE has a negative effect at high doses (0.75 and 0. 50%) on rat liver tissue (32). Wang et al. (33) showed that administration of OLE (250, 500 and 1000 mg/kg) improves apoptosis ratio on lead-induced damage in brain cortex. Unlike our data, they demonstrated that the highest dose of OLE (1000 mg/kg) was the most effective. However, further molecular and antioxidant studies are needed in order to determine the exact mechanism underlying the effects of different doses of OLE on BU-induced toxicity. 

Traditional herbs usage for therapeutic purposes has never guaranteed the safety of these plant. The liver is one of the most important organs in the uptake, metabolism, and elimination of drugs; therefore, in this study, in order to monitor possible toxicity of different doses of this extract on the liver, liver enzymes were studied. In the present study, in line with toxic effects of OLE 750 mg/kg on the testis, an increase in liver enzyme levels in rats that received OLE 750 mg/kg, indicated liver damage (34) and suggested that this dose of OLE could be toxic. In addition, inflammation was observed in the liver of rats that received OLE 750 mg/kg. However, such changes are not seen following administration of other doses of OLE.

Al-Attar and Abu Zeid (14) reported that diazinon could increase liver enzyme levels; in addition, administration of OLE 400 mg/kg to male mice exposed to diazinon could decline the levels of the mentioned enzymes but it did not reach the levels of the controls. In the current study, no statistically significant difference was observed in liver enzymes levels between rats which received OLE 500 mg/kg for five weeks and the control and BU groups. However, some studies, in agreement with our data, showed that administration of olive extract at high doses may be associated with liver damage. Omer-Sawsan showed that OLE at high concentrations (0.9%) increased ALP, LDH, and AST enzymes after seven and 14 weeks of treatment. They showed that this effect of OLE is dose-dependent (35). Arantes-Rodrigues et al. (32) demonstrated that different concentrations OLE (0.5 and 0.75% m/m) may have negative effects on liver function and even cause liver cirrhosis.

Previous studies demonstrated that the level of testosterone is different between control and BU groups (13). On the Contrary, in this study, there was no significant difference in total testosterone level among all groups. However, in this study, destruction rate of Leydig cells was higher in BU-treated than the control testes, while the number of Leydig cells increased significantly by all three doses OLE compared to BU-exposed testes. On the basis of our findings, we cannot attribute increased Leydig cell count in OLE-treated rats to the unchanged testosterone level.

## Conclusion

This study, for the first time, showed that administration of two doses of OLE (250 and 500 mg/kg), to Wistar rats could improve BU-impaired spermatogenesis and sperm quality without inducing liver damage. However, OLE 750 mg/kg not only had no ameliorating effect on testis and sperm parameters in BU-exposed animals, but also increased apoptosis rate in the germ cell and enhanced liver enzymes that indicate a liver damage and probable dysfunctions of other important organs.
